# Preliminary evidence of the effects of a novel antioxidant supplement in reducing oxidative stress in patients with senile cataract

**DOI:** 10.1007/s10792-023-02728-9

**Published:** 2023-06-20

**Authors:** Noriko Himori, Hiroshi Kunikata, Toru Nakazawa

**Affiliations:** 1 Department of Ophthalmology, Tohoku University Graduate School of Medicine, 1-1 Seiryo-cho Aoba-ku Sendai Miyagi, Sendai, 980-8574 Japan; 2 Department of Aging Vision Healthcare, Tohoku University Graduate School of Biomedical Engineering, Sendai, Japan; 3 Department of Retinal Disease Control, Tohoku University Graduate School of Medicine, Sendai, Japan; 4 Department of Ophthalmic Imaging and Information Analytics, Tohoku University Graduate School of Medicine, Sendai, Japan; 5 Department of Advanced Ophthalmic Medicine, Tohoku University Graduate School of Medicine, Sendai, Japan

**Keywords:** Oxidative stress, Supplement, Cataract, MDA

Dear Editor,

Systemic oxidative stress may be a key factor in the development of cataract. Moreover, past findings support the idea that antioxidant therapy may be effective for cataract patients. Chylack et al. [1] found that an oral antioxidant slightly slowed cataract progression. Maraini et al. [2] found that subjects who received multivitamin/mineral supplementation had a 36% lower prevalence of nuclear cataracts. Additionally, Maekawa et al. [3] reported that three food-derived compounds, hesperidin, *Tamarindus indica*, and crocetin, had a protective effect in a primary culture of retinal cells under oxidative stress. Hesperidin is effective in reducing apoptosis, oxidative stress and inflammation. Maekawa confirmed that hesperidin was effective in vivo in mice, reducing oxidative stress and preventing retinal ganglion cell death caused by NMDA-induced excitotoxicity [3]. Here, we investigated the effects of a novel supplement containing the same three antioxidants (hesperidin, crocetin, and *Tamarindus indica*) on markers of oxidative stress in patients with cataract.

This study had a prospective, single arm design. Twenty Japanese subjects with cataract were recruited (average age; 63.2 ± 7.2 years, male/female = 6/14, Table 1). Subjects were included if they were in overall good health, were between 49 and 74 years old, had cataract confirmed in one or both eyes by an ophthalmology specialist, and had a body mass index below 26 kg/m^2^. Subjects were excluded if they had severe systemic disease, including cancer, hyperthyroidism, and autoimmune disease. Patients with diabetes, hyperlipidemia, hypertension, or a current smoking habit were not excluded, but the condition was recorded. We asked them to refrain from the use of vitamin or carotenoid supplements for 2 weeks before the study. The subjects took 4 tablets together with ample water twice a day for 8 weeks, stopped the treatment, and were then followed for an additional 8 weeks. We calculated the effective dose for human subjects based on findings in a report by Maekawa et al. [3, 4]. The subjects were examined at four-week intervals, for a total of 5 examinations. Clinical laboratory data included venous blood levels of malondialdehyde (MDA), an end product of free radical reactions in membrane fatty acids that serves as an oxidative stress marker. Clinical parameters were also recorded. Comparisons used a one-way analysis of variance (ANOVA) followed by Dunnett’s test.

**Table 1:** Characteristics of cataract patients

Demographics	All
Number	20
Age (years)	59.1 ± 9.0
Sex (male:female)	6:14
Visual acuity (logMAR)	0.02 ± 0.23
IOP (mmHg)	14.71 ± 2.61
BMI (kg/m2)	22.36 ± 2.22
Diabetes (%)	0 (0.00)
Hypertension (%)	6 (30.00)
Current smoker (%)	2 (10.00)
Hyperlipidemia (%)	3 (15.00)

**Fig. 1:**
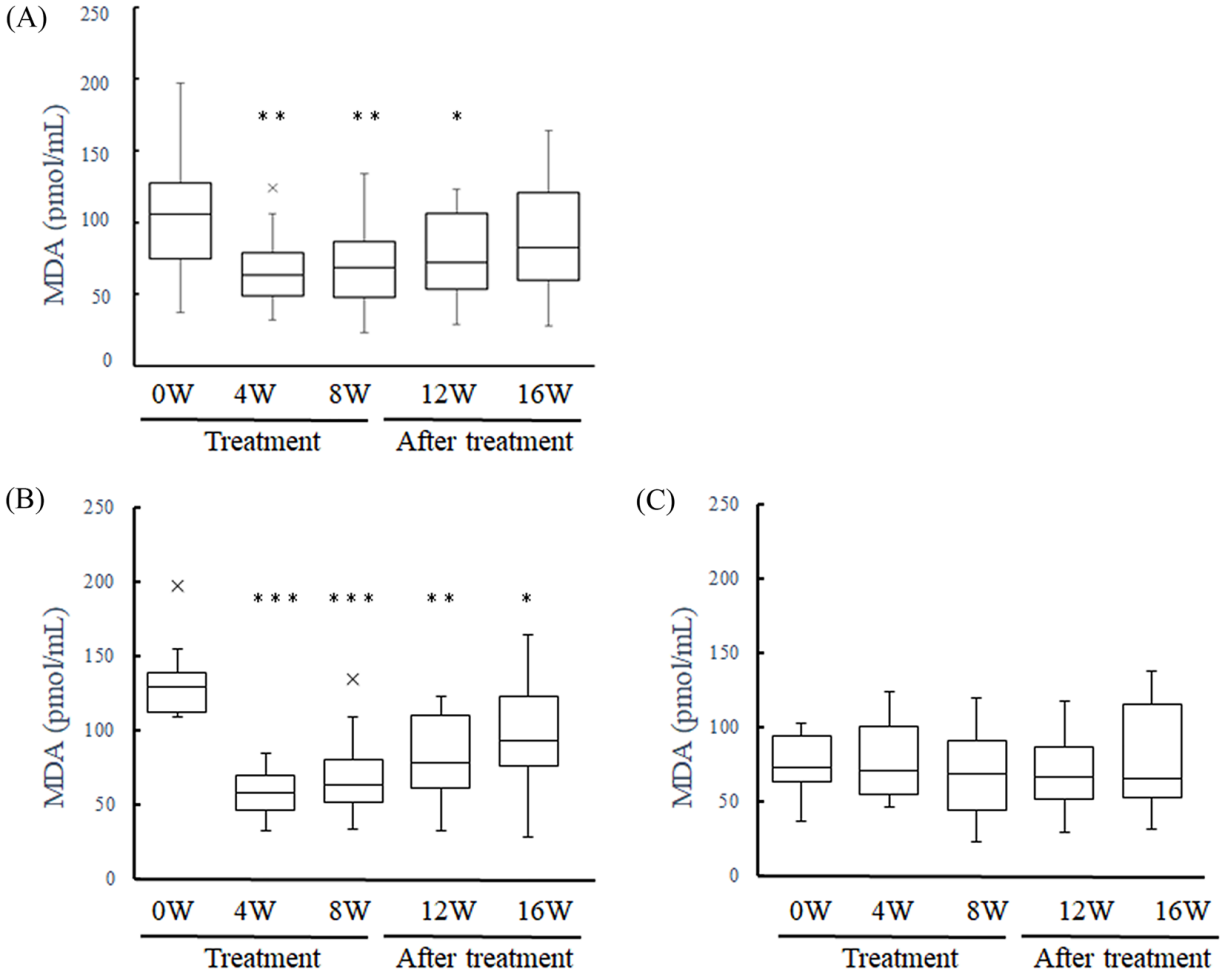
Changes in MDA level after antioxidant treatment. **A** MDA significantly increased at weeks 4, 8, and 12. **B** MDA significantly decreased at weeks 4, 8, 12, and 16 in patients with higher oxidative stress. **C** MDA did not significantly change after treatment in patients with lower oxidative stress.*p < 0.05, **p < 0.01, ***p < 0.001

In the overall group of 20 patients, the MDA level was significantly reduced at weeks 4, 8, and 12 (*P* < 0.01, *P* < 0.01,* P* = 0.04, Fig. 1A). We also divided the patients into high and low oxidative stress groups, based on whether their initial MDA level was above or below the group average of 103.25 pmol/mL (each group n = 10). In the high-stress group, the MDA level was significantly reduced at weeks 4, 8, 12, and 16 (*P* < 0.001, *P* < 0.001, *P* < 0.01, *P* < 0.05, Fig. 1B). In the low-stress group, the MDA level showed no significant changes (Fig. 1C). There were no supplement-related adverse events or abnormal results from blood testing in any of the patients.

Our study found that an 8-week oral course of antioxidant supplementation effectively reduced a biomarker of oxidative stress in patients with initially high oxidative stress. Hayashi et al. compared the total amount of hydroperoxides in the aqueous humor before and after supplementation with an antioxidant, Ocuvite Lutein [5], and found that hydroperoxides decreased in female, but not in male, subjects. That study suggested that it might be possible to inhibit oxidative stress in the aqueous humor and the lens epithelium. Lutein has been investigated by several in vitro studies, and lutein supplementation in lens epithelial cells has also been reported to decrease protein oxidation, lipid peroxidation, and DNA damage induced by H_2_O_2_ [6]. Based on these data, we speculate that decreasing general oxidative stress with supplementation might also reduce local oxidative stress in the aqueous humor, potentially delaying cataract progression. It is thought that oxidative stress is a risk factor for other ocular diseases, such as dry eye and age-related macular degeneration. Therefore, taking the supplement object of the current study could help to reduce general oxidative stress, having a potential effect on the prevention or slowdown of these conditions.

Our study has limitations. First, it lacked a placebo arm, which is needed to prove the efficacy of any supplement. Second, the sample size was relatively small. There were only 20 patients who were available for a follow-up of 16 weeks during the recruitment period (March 2018–October 2019). Despite this, the reduction in general oxidative stress with oral administration of our supplement was statistically significant, leading us to consider that the sample size was sufficient. Finally, only one oxidative stress marker was used in this study. Thus, we plan to conduct further studies with a randomized, double-blind, placebo-controlled design using a greater number of oxidative stress markers.

This study shows that supplementation with an oral antioxidant was an effective treatment in patients with cataract and high oxidative stress. Thus, supplementation with antioxidants may be a promising new way of ameliorating diseases caused by systemic oxidative stress, and may become part of individualized medicine.
